# Celiac Artery Dissection in a Young Smoker

**DOI:** 10.7759/cureus.4047

**Published:** 2019-02-11

**Authors:** Hector H Gonzalez, Levonti Ohanisian, Angel E Sanchez, Vlad Voin, Javier Ricardo

**Affiliations:** 1 Internal Medicine, Florida Atlantic University Charles E. Schmidt College of Medicine, Boca Raton, USA; 2 Miscellaneous, Florida International University, Miami, USA

**Keywords:** celiac artery dissection, ct angiography

## Abstract

Isolated dissection of the celiac artery (IDCA) is a rare phenomenon with unclear pathogenesis. Although several risk factors have been attributed, it is essential for clinicians to maintain this diagnosis in the list of differentials to ensure prompt implementation of diagnostic and treatment modalities. We present the case of a 37-year-old smoking male who presented with upper abdominal pain radiating to the left upper quadrant with associated nausea/vomiting, diaphoresis, and diarrhea.

## Introduction

Celiac artery dissection is rare and the true incidence is unknown. A review of 450 cases of spontaneous mesenteric artery dissection (SMAD) showed that about half of the cases affected the celiac artery (200) and the other half affected the superior mesenteric artery (250) [1]. The celiac artery gives rise to three major arteries; left gastric artery, common hepatic artery, and splenic artery, which makes it an important artery supplying blood to multiple vital organs [2]. Identifying celiac artery dissection early and treating it appropriately is thus vital to the patient’s morbidity and mortality. We present a case of spontaneous celiac artery dissection in a relatively young and healthy male.

## Case presentation

**Figure 1:**
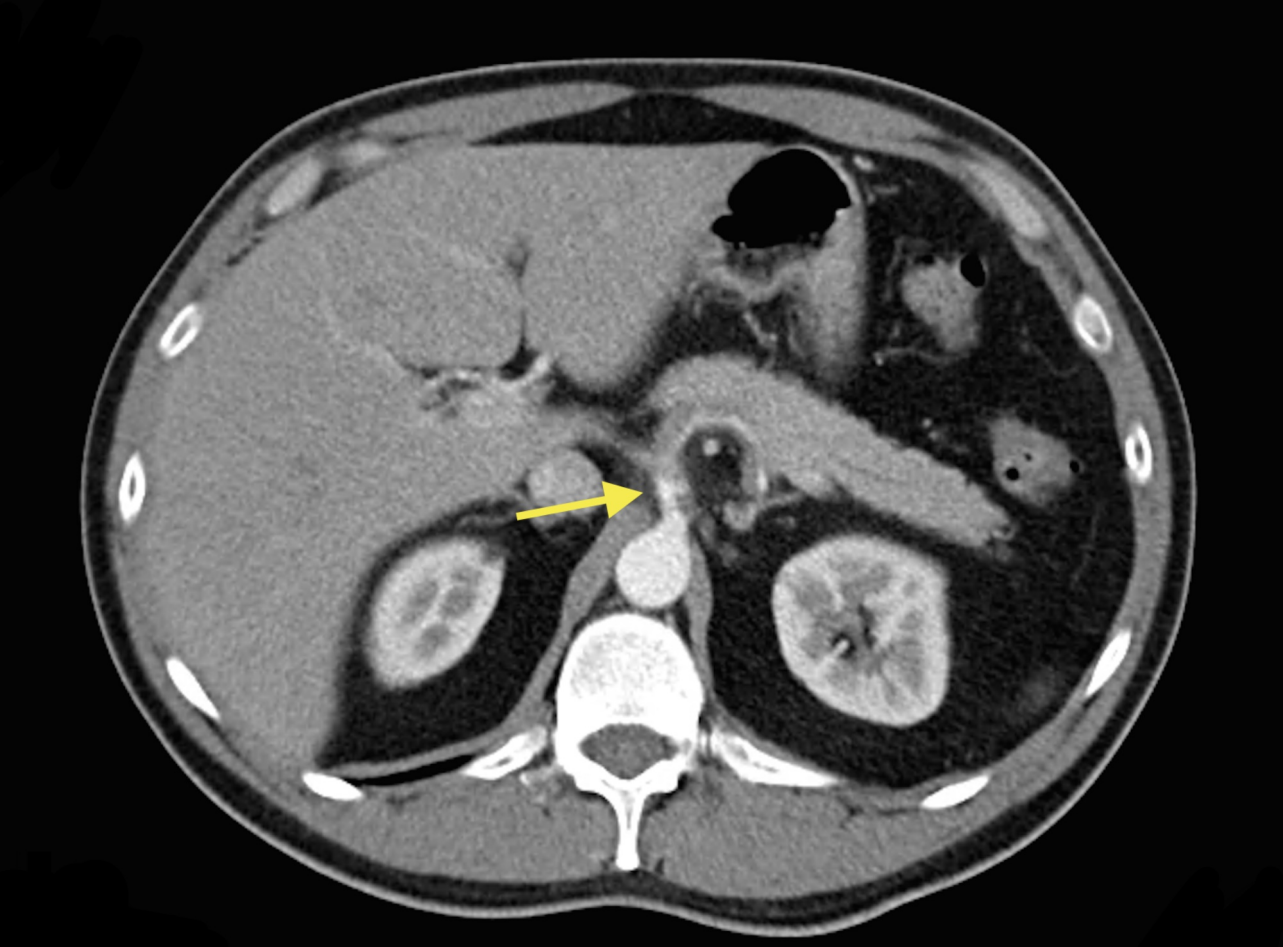
Computerized tomography (CT) of the abdomen and pelvis with contrast showed focal dissection of the celiac artery (arrow)

The patient is a 37-year-old male with a past medical history of smoking two packs per day since the age of 13. He presented to the emergency department with a chief complaint of sharp, epigastric pain radiating to left upper quadrant, 10/10 in intensity, associated with non-bloody vomiting and diarrhea. The patient also endorsed having diaphoresis but otherwise denied fevers/chills, chest pain, shortness of breath, hematochezia/melena. On physical exam, he was tachycardic and abdominal exam showed tenderness on palpation of the epigastric area and right upper quadrant. Laboratory data revealed hemoglobin of 18 g/dL, hematocrit 49.6%, platelets 254,000/mm^3^, prothrombin time 11.1 seconds, partial thromboplastin time 26.4 seconds, international normalized ratio (INR) 1.1, white blood cell (WBC) 18.8 K/µl. Liver function tests showed alkaline phosphatase (ALP) 73 U/L, alanine aminotransferase (ALT) 24 U/L, aspartate aminotransferase (AST) 25 U/L. A computerized tomography (CT) of the abdomen and pelvis with contrast showed focal dissection of the celiac artery which extended out to the splenic artery and resulted in minimal flow through the splenic artery. Asymmetric enhancement of the spleen was seen which may have been related to diminished flow or areas of splenic infarction (Figure 1).

**Figure 2:**
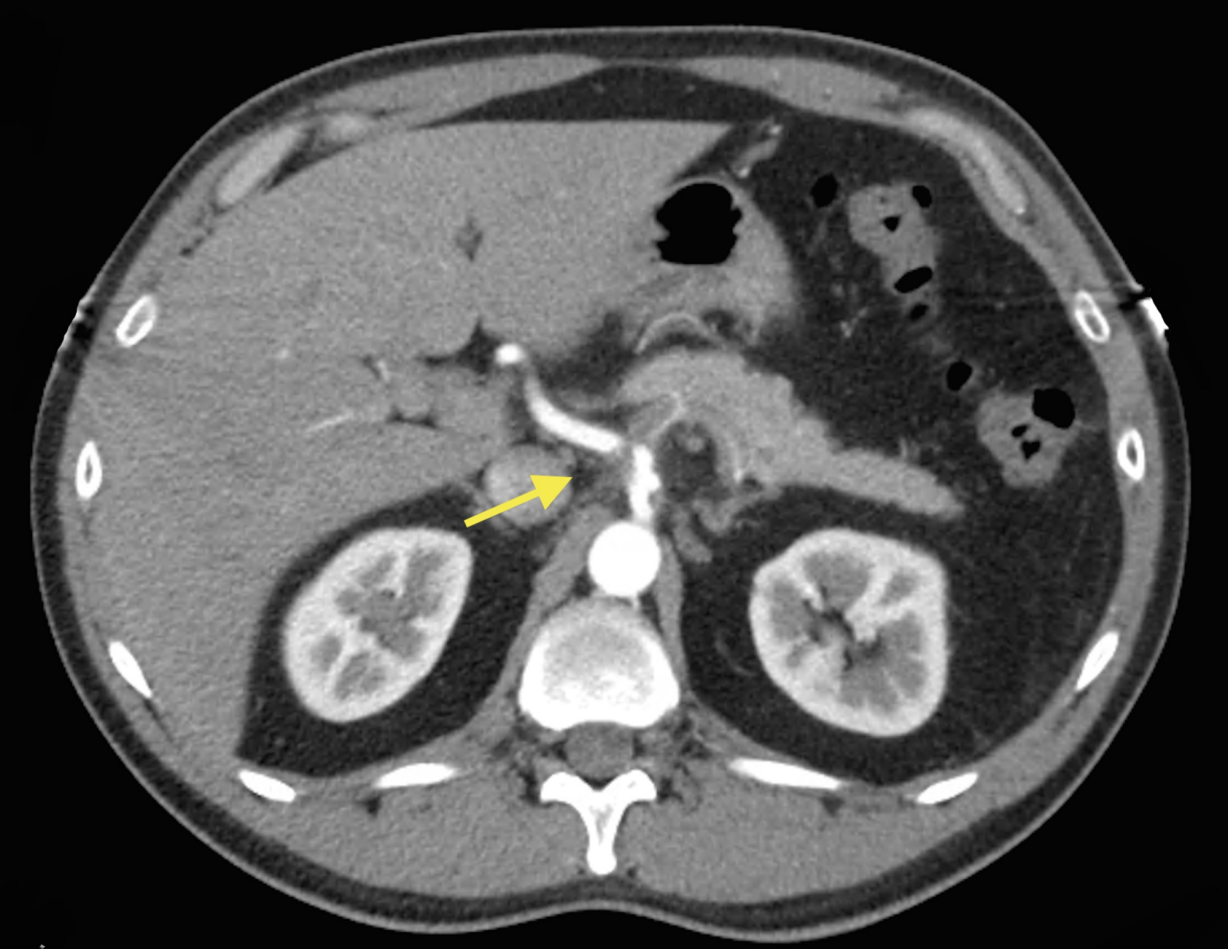
Computed tomography angiography (CTA) of the abdomen and pelvis depicting stenosis within the proximal celiac trunk of less than 50% with greater stenosis at the celiac bifurcation (arrow)

**Figure 3:**
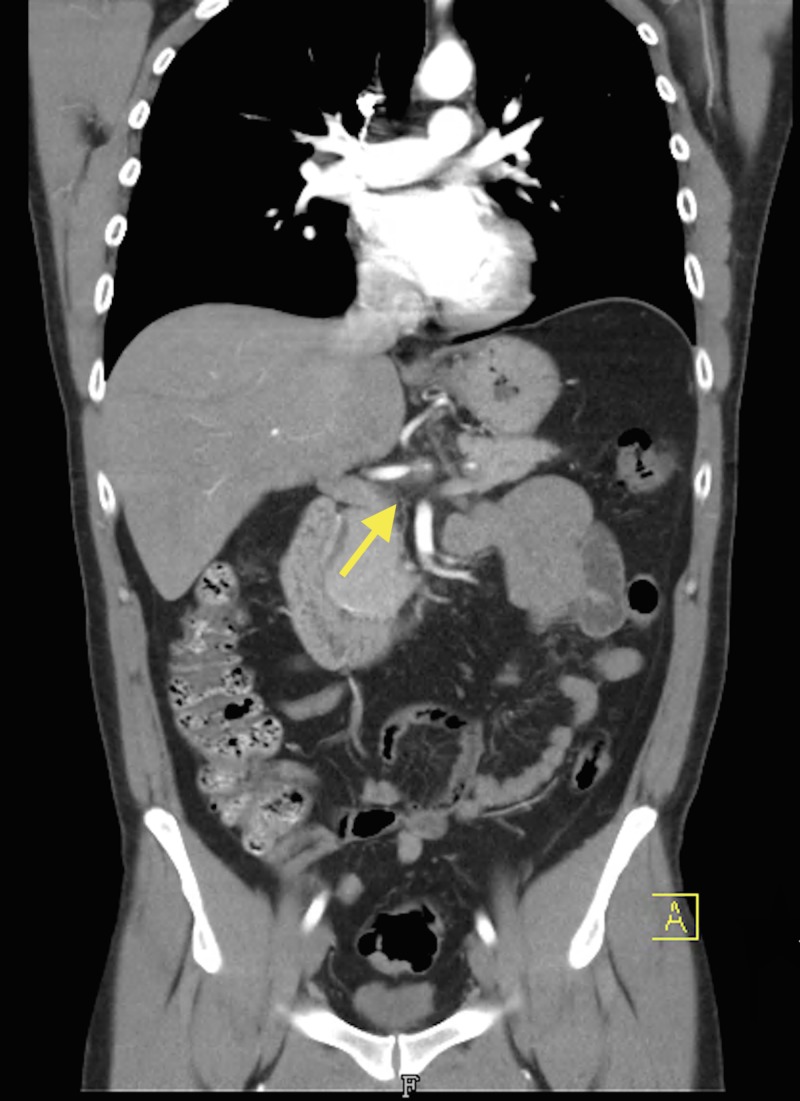
Coronal computed tomography angiography (CTA) of the abdomen and pelvis depicting celiac artery dissection with proximal hepatic artery stenosis (arrow)

**Figure 4:**
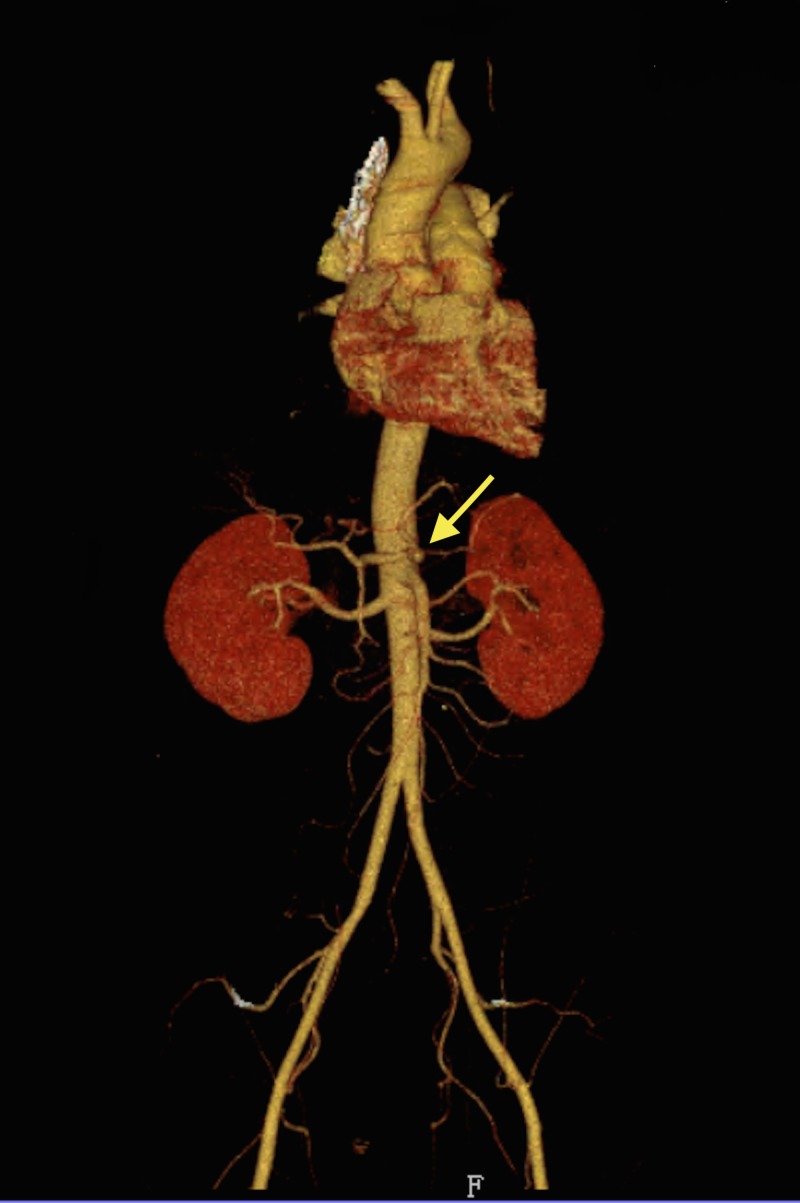
Computer tomographic (CT) three-dimensional reconstruction showing celiac artery dissection with proximal hepatic stenosis (arrow)

Vascular surgery was consulted and he was placed on a heparin drip. Magnetic resonance angiogram (MRA) of the abdomen reported an abnormal celiac axis with an irregular narrowed appearance consistent with suspected dissection as seen on CT scan. There was a stenosis several centimeters after the origin likely at the junction between the celiac trunk and the common hepatic artery. No flow was detected in the splenic artery on magnetic resonance imaging (MRI) of the abdomen. Computed tomography angiography (CTA) of the abdomen and pelvis showed stenosis within the proximal celiac trunk of less than 50% with greater stenosis at the celiac bifurcation. The stenosis at the origin of the hepatic artery was approximately 75% and the hepatic artery distal to this site was normal in appearance (Figures 2-4).

The stenosis at the origin of the splenic artery was at least 75% and continued throughout the remainder of the splenic artery. Whether the findings of the splenic artery represented stenosis secondary to thrombus or dissection was not definitively determined given the small size of the vessel; however, no dissection was seen within the aorta. There was also an accessory left renal artery without stenosis of the renal arteries bilaterally, and an area of splenic infarct.

The patient underwent percutaneous balloon angioplasty of the hepatic artery and mechanical suction thrombectomy of the common hepatic artery by vascular surgery. While hospitalized, the patient was evaluated by hematology and a hypercoagulable workup was performed with no significant findings including factor V levels, antiphospholipid antibodies, and anticardiolipin antibodies. The patient was discharged on coumadin 5 mg daily with an INR of 2.9 and was told to continue taking labetalol 200 mg orally twice a day for blood pressure control.

## Discussion

Isolated dissection of the celiac artery (IDCA) is a rare pathology describing dissection of the celiac artery with the absence of an aortic dissection [3]. Dissection of the celiac artery is one manifestation of spontaneous mesenteric arterial dissection which may include dissection of the superior mesenteric artery and the inferior mesenteric artery. SMAD presents in patients who are in their fifth or sixth decade of life [4]. Although the exact etiology and pathogenesis remain unknown, risk factors may include hypertension, smoking, atherosclerosis, abdominal surgery, trauma, fibromuscular dysplasia, connective tissue disease, pregnancy, vasculitis, tumor, and cystic medial necrosis [3]. Interestingly, the patient presented in this case evidenced only one of the many risk factors proposed – smoking. Additionally, the patient was well younger than patients who had been previously reported with SMAD.

Clinical manifestations of IDCA depend on disease severity and extent. This includes the dissection-affected range as well as the presence of secondary vascular rupture or bleeding present. Despite this variability, most patients present with the chief complaint of sudden upper abdominal pain associated with nausea, vomiting, and dizziness with pain radiating to the back or shoulder [3]. Our case adds radiation, to the left upper quadrant, as an additional location of radiating pain. Reports of patients presenting with associated symptoms of decreased appetite, postprandial pain, chest pain, weight loss, and intestinal colic have also been observed [3]. Our case adds non-bloody diarrhea and diaphoresis as additional associated symptoms.

Diagnosis of IDCA is made with imaging. CTA is considered the diagnostic test of choice, although other imaging modalities such as ultrasonography and conventional angiography may be used [5]. CTA provides sufficient imaging of the mesenteric vasculature regardless of body mass and anatomic abnormalities which would make ultrasonography challenging to use. Importantly, CTA has the ability to provide a three-dimensional view of luminal borders and highlight extraluminal disease [6].

Treatment can either be conservative or endovascular therapy. A systematic review and meta-analysis by Wang et al. investigating the clinical course of IDCA, concluded that 8% of patients who were initially managed conservatively required secondary intervention [4]. The authors concluded that conservative treatment of celiac artery dissection is safe; however, provided the caveat that patients with high occurrence of late secondary intervention may require closer monitoring [4]. There has been no expert consensus on the best approach to treatment for patients with IDCA. However, intravascular methods have resulted in shorter hospital stays and high cure rates and have therefore been adopted by several institutions as the treatment of choice [3]. If patients choose to undergo conservative treatment, regular CTA must be done to monitor recurrence or increased involvement of disease [5]. Endovascular treatment has been used to treat celiac artery dissections previously including the use of stenting, balloon fenestration, and transcatheter embolization with coils [7-9]. Our patient underwent endovascular therapy to treat the lesion initially with a mechanical suction thrombectomy device. Suction thrombectomy was performed along with subsequent balloon angioplasty resulting in significantly improved blood flow.

Due to the severe consequences of IDCA reported such as splenic infarction, intraperitoneal hemorrhage, and intestinal ischemia [10-12] it is imperative to include it in the differential diagnosis of patients presenting with upper abdominal pain with associated nausea, vomiting or dizziness, despite the already large list of possible diagnoses. Our case highlights the importance of including IDCA in the differential diagnosis despite the presence of only one risk factor. Additionally, our patient was in his third decade of life while IDCA has most commonly affected patients in their fifth and sixth decade of life [4]. We present the case of a 37-year-old male with only one risk factor who presented with IDCA; therefore, this diagnosis should remain on the differential diagnosis even in a younger population. Furthermore, this case may shed light on the importance of smoking as a risk factor for celiac artery dissection. While smoking is currently lumped together with a multitude of risk factors, future case reports and reviews of the literature are needed to examine the relative risk of smoking on celiac artery dissection.

## Conclusions

IDCA is a rare pathology with unclear etiology and pathogenesis; however, several risk factors have been associated with it. Diagnosis is established by CTA and treatment may be either conservative or involve an endovascular approach. Our case demonstrates the importance of including celiac artery dissection in the differential diagnosis of young patients who present with upper abdominal pain as well as adding the left upper quadrant as a new location for pain radiation with the associated symptoms of diaphoresis and non-bloody diarrhea. Lastly, since smoking was the only risk factor present in our patient, it raises the question of whether smoking has a higher relative risk for celiac artery dissection than other risk factors.
